# Assessing family function: older adults vs. care nurses: a cross-sectional comparative study

**DOI:** 10.1186/s12889-024-18809-y

**Published:** 2024-05-17

**Authors:** Mei-Wen Wang, Ya-Mei Chen

**Affiliations:** 1 Department of Family Medicine, Keelung Chang Gung Memorial Hospital, Keelung, Taiwan R.O.C.; 2 College of Public Health, National Taiwan University, Taipei, Taiwan R.O.C.

**Keywords:** Older adults, Geriatric, Home-bound, Home nursing care, General function of family assessment device

## Abstract

**Background:**

This study aimed to assess family function in home care for older adults. Understanding family dynamics is essential for providing quality care to older adults choosing to age in place.

**Methods:**

In a cross-sectional study, 53 patients aged 65 or older receiving home care were evaluated, along with four home care nurses. The General Function of Family Assessment Device (FAD-GF) was used for self-assessment to examine family resources.

**Results:**

Only 5.7% of older adults reported good family function. Strong correlations were found between assessments by nurses and older adults. Among the six aspects of family function, “problem solving,” “communication,” “affective responsiveness,” and the overall results showed no disparities between the evaluations of older adults and nurses.

**Conclusions:**

Home care nurses can effectively assess family function using the FAD-GF, particularly after six months of care. This assessment can help identify family issues and enhance home care quality through nurse training in FAD-GF application.

## Background

Older adults are defined as people older than 65 years old [36]. The proportion of older adults in Taiwan increased from 11.5 to 13.33% from 2013 to 2017 and it was estimated that older adults would make up more than 20% in 2026 [9]. Health and care in older adults will be a challenge to social welfare and policy. Most older adults face a multitude of physiological and psychological complexities in terms of health and daily life functioning [23] and caring for them demands high-quality care and increased attention [14]. From numerous disabilities and disorders, these difficulties make it more challenging for older adults to complete questionnaires or participate in investigations of their nutrition, family support, family function, or the quality of care. However, compared to having family members assist in the assessment, we need individuals who are more objective and have a better understanding of the patient’s condition to help assess their family functioning, such as home care nurses who provide long-term care for the older adults.

Home care that includes any professional support services for older adults, provides them with the opportunity to stay at home and age in place. In Taiwan, the government administration has multiple guidelines to promote healthy aging, however, there are still many challenges to remaining active and healthy while aging. In Taiwan, caring for family members who are sick or weak is a cultural value. Family members play an important role in relationships that affects the health of the aged and the quality of their care [10, 29]. Family function refers to both the social and structural properties of the global family environment, which includes interactions and relationships, adaptability, organization, and the quality of communication within the family [1, 27, 29]. Good family function decreases self-negligence of the older adults and improves their social ability and their health [1, 27, 34]. Just like during the COVID-19 pandemic, elderly individuals from families with poor functioning tend to feel depressed, lonely, and may experience some psychological issues [24, 25]. However, most older adults individuals in long-term care are likely to experience family dysfunction more [8].

Development of home care and community care systems is essential to facilitate aging in place, however, the need for and cost of long-term care increases every year [21]. It is estimated that around 40,000 older adults residing at home in Taiwan suffers from severe dysfunction and 270,000 have mild impairment and these statistics are likely predicted to double in 40 years [4]. Home care is the future direction for older adult care [6, 13, 27, 32] and good family function is essential to make the home care system effective [15, 30]. Home care nurses provide connections with the patient, the caring system, and the family members as well as to identify and prioritize any problems [13]. They may visit a patient once or twice a month with daily telephone calls and are qualified to evaluate a patient’s family function, however, the time the nurse has spent caring for the patient may be a factor affecting the results of the evaluation [35].

The assessment of family functioning plays a pivotal role in influencing the overall health and quality of care for the older population. Its significance lies in several key aspects. First and foremost, robust family functioning provides a stable social support network for seniors, which serves to reduce feelings of loneliness, enhance psychological well-being, and mitigate psychological issues such as depression and anxiety [18]. Additionally, it ensures that older adults receive appropriate care and support in their daily lives. Furthermore, the evaluation of family functioning is intricately linked to the quality of life experienced by older adults [18]. When family relationships and interactions are positively healthy, seniors are more likely to enjoy a meaningful life, engage in social activities, and maintain physical and mental well-being [20].

Moreover, the assessment of family functioning also takes into consideration not only the patient themselves but can also provide early awareness of the emotional state of the caregivers [26]. This is crucial in promoting healthy aging and improving the quality of long-term care. In summary, assessing family functioning is an essential component of public health efforts aimed at promoting healthy aging and enhancing the quality of long-term care for the older adults.

In a previous study, the Family Adaptation Partnership Growth Affection Resolve (APGAR) score was used and judged to be appropriate for the evaluation of family function of older adults by home care nurses who had cared for the older adults for at least 6 months [35]. However, the questionnaire could only reveal family dysfunction, and it was difficult to determine which aspects should be further evaluated to help improve family function [28, 33]. As we have known in the past, the physiological and psychological health of the older adults is complex and easily influenced [3]. Therefore, a single assessment of family functioning may not be sufficient to help detect and address problems early. We need a more comprehensive assessment of family functioning to assist in improving the health of the older adults [17, 22]. Therefore, the purpose of this study was to assess whether home nurses can assist in assessing the family function of the care receivers, determining the structural, sociological, and emotional problems using the Family Assessment Device (FAD) self-assessment test. Our hypothesis was that the home nurses are capable in helping to evaluate their patients’ family function using FAD.

## Materials and methods

### Study Design

This study was carried out in northern Taiwan and was reviewed and approved by the institutional review board, with written consent obtained from the patients. This was a cross-sectional and quantitative study with a descriptive correlational design. The theoretical approach used was the subscale, General Function of Family Assessment Device (FAD-GF).

### Participants

The participants were recruited from the home care department at the Chang Gung Hospital in northern Taiwan. The participants were members of the home care program offered by the government health units, which provided care by well-trained nurses at a regional hospital in Keelung. All included patients are those who were eligible in this home care program. The inclusion criteria were one) participants has to be over the age of 65 that was in a home care program with a home care nurse and two) a score below 60 in Barthel Index (used to assess functional independence). If participants had severe impairments of hearing, language, or comprehension, dementia, psychiatric disease, or any disorienting conditions, that cannot complete or comprehend the FAD, they were excluded. 324 older adults who were cared for in the home care department were recruited; however, only 53 met the inclusion criteria. Home care nurses that were paired with their individual older adult in home care who visited their home also recruited and participated in this study. A total of four home care nurses who worked in the same hospital had cared for the older adults and their families from their regular visits of once per two weeks to three months were included.

### Data collection procedures

Data were collected from June 2012 to January 2013 through individual interviews with the older adults without other family members present and with home care nurses separately. Written informed consent was obtained from both the home care nurses and the older adults after the understanding of this research’s purpose and procedures explained at the beginning of each recruitment. Only one research assistant (RA) was assigned in this study. The RA first gave the home care nurses FAD questionnaires to complete before visiting their older adults. Later the same day, the questionnaire was completed by the older adults alone or with the aid of the RA. The RA could only help when older adults could not see or read the questions and would only help to read out the questions and to record the answers if necessary.

### Instruments

The McMaster FAD, developed by Nathan in 1983, is a common and useful self-report scale to measure an individual’s perceptions of a family function [11, 16]. The questionnaire is made up of 60 items, with seven components, including six aspects of family function (“problem solving,” “communication,” “roles,” “affective responsiveness,” “affective involvement,” and “behavioral control”) and an evaluation of general function subscale. The items consist of general statements about families. The general function part has 12 items, consisting of two items for each of the six aspects. Each item is scored on a scale from 1 to 4 (strongly disagree, disagree, agree, and strongly agree). Each respondent has to answer how well the statement in each item represents the family. To obtain the overall mean family score, the scores were averaged in the end. The cutoff point was 2. A final score of greater than 2 but less than 3 indicates “moderate family dysfunction”. A final score of greater than 3 indicates “family dysfunction” [11, 19]. The score on the general function subscale is correlated with the total score [11].

The subscale of the general function part of the family assessment device (FAD-GF) was also used which was validated in 1985. Chinese version was translated in Hong Kong in 2002 [7, 19] and was yielded a Cronbach’s α between 0.78 and 0.86 [5, 12]. Poorer family function is defined as a score greater than 2 on the FAD-GF scale and better family function as a score less than 2 [11].

### Data analysis

All statistical analysis was collected using Statistical Package for the Social Sciences. The data were analyzed by the paired *t*-test and the McNemar–Bowker test. 95% confidence intervals were calculated and a *P*-value < 0.05 was considered to indicate a statistically significant difference. The reliability of the questionnaire was tested among nurses, producing a standard Cronbach’s alpha of 0.943. All the questions were essential and the factor analysis was carried out. The Kaiser–Meyer–Olkin value was 0.888, and the Bartlett sphericity test showed the *P*-value to be < 0.005. Principal component analysis was also performed, and the cumulative percentage of the extraction sums of the total variances explained squared loadings was 71.167%. The results showed that the questionnaire could also be used for evaluation by the nurses.

## Results

Table 1 presents the baseline characteristics of 53 older adults that were included in this study. The average age was 79.2 (ranging from 65 to 92) years while the average caring time was 737.9 (ranging from 16 to 4072) days. In the distribution report of the family function evaluated by the older adults and the nurses, family function was categorized as “good”, “moderate”, or “dysfunction”. Older adults evaluated only 5.7% had good family function, 50.9% had moderate family function, and 43.4% had dysfunction family. Nurses evaluated 5.7%, 34.0%, and 60.3% respectively.

**Table 1:** Older adults’ characteristics

Characteristics	*n*	%
Sex		
Female	28	52.8
Male	25	47.2
Age(years)		
65–74	15	28.3
Above 75	38	71.7
Education		
Illiterate	18	34.0
Elementary school	24	48.3
Senior high school	5	9.4
High school	2	3.8
Bachelor’s degree	4	7.5
Length of caring time		
< 3 months	4	7.5
3–6 months	7	13.2
6–12 months	15	28.3
> 12 months	27	51.0

Estimates of family function by older adults are presented in Table 2. No female participant reported good family function. There were no significant relationships between the degree of family function and any of the variables. In Table 3, a paired *t*-test was used to examine the relationship between older adults’ and their home care nurses’ evaluation of family function according to caring time. The results showed that the nurses should take care of older adults for at least 6 months up to 12 months before using the questionnaire to evaluate the older adults’ family function. Because only those with caregiving experience exceeding three months were considered, there still exists a notable disparity between the assessments conducted by nurses and those by older adults (*P* = 0.038). In Table 4, there were no significant differences between the older adults’ and the nurses’ evaluation of the levels of family function when caring time was more than 6 months (*P* = 0.108 > 0.05, McNemar–Bowker test). There was a moderate to high correlation between the older adults’ and the nurses’ evaluation of family function when caring time was more than 6 months (*R* = 0.583, *P* < 0.001; Pearson’s product-moment correlation coefficient test). A kappa value of 0.431(*P* = 0.001) indicated that family function levels estimated by nurses showed no significant differences when compared by the older adult’s condition.

**Table 2:** Family functionality according to the variables of sex, age, level of education and the length of caring time by older adults’ estimation

Variables	FAD_General Function						Total		
	Dysfunction		Moderate		Good		n	%	
	n	%	n	%	n	%			P
Sex									0.168
Female	13	56.5	15	55.5	0	0.0	28	52.8	
Male	10	43.5	12	44.5	3	100.0	25	47.2	
Age (years)									0.605
65–74	8	34.8	6	22.2	1	33.3	15	28.3	
Above 75	15	65.2	21	77.8	2	66.7	38	71.7	
Education									0.950
Illiterate	9	39.1	8	29.6	1	33.3	18	34.0	
Elementary school	11	47.8	12	44.4	2	66.7	25	47.2	
Senior high school	1	4.3	3	11.1	0	0.0	4	7.5	
High school	1	4.3	1	3.7	0	0.0	2	3.8	
Bachelor’s degree	1	4.3	3	11.1	0	0.0	4	7.5	
Length of caring times									0.267
<3 months	2	8.7	2	7.4	0	0	4	7.5	
3–6 months	1	4.3	5	18.5	1	33.3	7	13.2	
6–12 months	4	17.4	10	37.0	1	33.3	15	28.3	
>12 months	16	69.6	10	37.0	1	33.3	27	50.9	

**Table 3:** Relationship between older adults’ and their home-care nurses’ evaluation of family functioning by paired-T test

						Older adults *Nurses					
	N		Mean(E) ± SD		Mean(N) ± SD	CI		R	P		
> 3 months	49		2.37 ± 0.60		2.55 ± 0.61		-0.35~-0.01	0.51*	0.038*		
> 6 months	42		2.43 ± 0.59		2.60 ± 0.59		-0.33 ~ 0.01	0.58*	0.051		
> 12 months	27		2.55 ± 0.58		2.63 ± 0.63		-0.26 ~ 0.11	0.69*	0.425		
total	53		2.83 ± 0.54		2.98 ± 0.57		-0.28~-0.15	0.62*	0.030*		

^*^P < 0.05

**Table 4:** Relationship between older adults’ and their home-care nurses’ evaluation of family functionality, caring time more than 6 months (*N* = 42)

		Older adults								*P*	
		Dysfunction		Moderate		Good					
		n	%	n	%	n	%				
Nurses	Dysfunction	18	95.0	9	45.0	0	0.0				
	Moderate	2	5.0	10	50.0	1	50.0				
	Good	0	0.0	1	5.0	1	50.0		0.108^†^		

^†^*P*-value of McNear-Bowker test

Regarding the six aspects of family function, problem scores for each aspect were separated to evaluate the relationship between older adults’ and their nurses’ estimations of family function in Table 5. Only the scores for “problem solving”, “communication,” “affective responsiveness,” and the total result did not differ between the older adults and their nurses.

**Table 5:** Scores over six components among Family Assessment Device of nurses and older adults they cared more than 6 months

	Older adults *Nurses					*p*
	Mean(E) ± SD	Mean(N) ± SD	CI	R		
Problem solving	3.07 ± 0.89	2.86 ± 0.73	-0.02 ~ 0.45	0.57*	0.07	
Communication	2.69 ± 0.78	2.88 ± 0.52	-0.45 ~ 0.07	0.21	0.15	
Roles	2.15 ± 0.95	2.85 ± 0.63	-0.96~-0.42	0.47*	0.00*	
Affective responsiveness	2.93 ± 0.64	3.01 ± 0.69	-0.32 ~ 0.16	0.33*	0.49	
Affective involvement	3.47 ± 0.76	3.07 ± 0.60	0.16 ~ 0.65	0.35*	0.002*	
Behavioral control	2.89 ± 0.84	3.19 ± 0.65	-0.55~-0.04	0.43*	0.002*	
total	2.43 ± 0.59	2.60 ± 0.59	-0.33 ~ 0.01	0.58*	0.051	

*CI = Confidence Interval

^*^P < 0.05

## Discussion

This study’s ultimate goal was to assess whether home nurses can assist in assessing the family function of the care receivers and to determine the potential structural, sociological, and emotional problems presented in families by estimating the family function by home care nurses and older adults using FAD. Previous study using Family APGAR could only reveal family dysfunction [35] and was required more information to determine which aspects should be further evaluated to help improve family function.

The family function estimate in this study was poorer than those reported in previous studies [8, 35]. Up to 94.3% of participants reported family dysfunction problems, a higher percentage than that in the institutional setting (80%), and higher than the result obtained for the home care system estimated by the Family APGAR (52%). This observation may be related to the method of evaluation. The FAD-GF uses a four-point scale, whereas the Family APGAR uses a three-point scale. The FAD-GF questions are more detailed, and peculiar questions are included to evaluate the real condition. In this study, most participants were in the moderate family at the dysfunction category, which is a vague designation. Only 43.4% of participants were classified as having true family dysfunction. This may indicate that the detailed information the questionnaire can provide is more important than the total score. The questionnaire may reveal more problems than other questionnaires used in clinical practice and help family physicians to evaluate and manage the problems more easily.

In this study, no specific category could affect the result of the evaluation, however, caring time was a variable that may be a factor affecting the results. It was necessary to perform further testing of the relationship between the estimated level of family function and caring time. In previous studies, nurses caring for older adults for more than six months could help the older adults to evaluate their family function. Even though the FAD-GF is a self-evaluation questionnaire, most patients cannot fill it out by themselves due to several difficulties. Therefore, longer caring times may be needed before nurses are qualified to help older adults to evaluate their family function by the FAD-GF. The questionnaire may ask for additional private information, which makes for a more detailed understanding of the family condition. Moreover, the necessity to observe the relationships and psychosocial problems in the family members may need further training or experience.

Our study mainly found out that the evaluation results of the family function conducted by older adults and nurses were moderately correlated. This meant that the home care nurses may represent the older adults they cared for when evaluating their family function by FAD-GF. Previously, the FAD was for aged over 12 years [11, 19]. The general function subscale could be used to screen for family function because of the high correlation with the total score on the FAD [2]. However, it is still a self-assessment questionnaire, which is difficult for older adults with poor visual acuity and lower educational level to complete. Moreover, some older adults may suffer from illnesses or diseases that cause them to have difficulty expressing their feelings or problems. This study shows that home care nurses can represent older adults in evaluating family function. Although the FAD-GF may require a longer caring time to assess in comparison with the Family APGAR, this questionnaire can nonetheless obtain much more detailed information useful in family health care, enabling family physicians to help older adults earlier and offer appropriate interventions when conducting home visits. It is most useful forth evaluation of “problem solving,” “communication,” and “affective responsiveness” and when used as a screening tool. With regard to the aspects of “roles,” “affective involvement,” and “behavioral control,” the results of estimations by nurses and older adults were significantly different. These three aspects may possibly be difficult for people outside of the family to understand, feel, or change. Even in this condition, family physicians could still find out the level of family dysfunction from the total score. If there are problems in “problem solving,” “communication,” or “affective responsiveness,” doctors can be involved and help with treatment immediately, therefore, are easier for them to be involved in these three aspects and to address related problems.

In past research, it was found that understanding family functioning and composition is crucial for home care among the elderly population [31]. The real-time assessment of family functioning holds significant value in academic research, clinical practice, and the development of care plans within the field of public health [11]. Therefore, the real-time assessment of family functioning is an important topic in the training of home care or medical personnel, within the field of public health. It aids in the early identification of issues, personalized care, improvement in the quality of care [26], and exerts a positive influence on academic and professional training.

This study had some limitations, including a lack on family composition, religion, and income data. Family composition refers to family members who live together, but many older adults lived alone in Taiwan. Their family members may live nearby, however most caregivers are not relatives of the older adults and may live together under private hired employment relationship. Religion may also affect the behavior of the family, which may also affect the family function results. The effect of religion was difficult to estimate, because most older adults find it difficult to join in religious activities outside of their homes. Economic status was also difficult to assess as it could affect the quality of care. This research was carried out in the northern Taiwan area where the average income was the lowest [37]. These limitations make it difficult to compare our results with those from other countries. Furthermore, the years of experience and age of home care nurses may indeed influence the results; however, as there were only four nurses in this study, assessment outcomes were based solely on caregiving time. If more data could be collected in the future, additional variables could be incorporated into the study for discussion. Lastly, the number of participants was small. Many older adults had chronic disease, cognitive impairment, or other diseases and/or disorders that required attention from home care nurses, which was difficult to include them.

## Conclusions

This study demonstrated the suitability of the general function subscale of the FAD as a way to evaluate the family function of older adults. Home care nurses could help to assess the family function of older adults if they had been caring for more than 6 months. As a result, older adults with moderate to poor family dysfunction could receive more attention from nurses, family physicians, and the home care staff. When screening for family function, the FAD-GF may provide more information. The results may enable family physicians to identify family problems more easily and earlier, help to improve the quality of life and health of older adults, and encourage the government to consider evaluation of the family function of older adults as one of the most important items when setting policy for long-term health care. Training home care nurses to utilize FAD-GF for conducting home functional assessments and identifying family care issues early may contribute to enhancing the quality of home care.

## Data Availability

The datasets used and/or analysed during the current study are available from the corresponding author on reasonable request.
